# Wafer-Level Self-Packaging Design and Fabrication of MEMS Capacitive Pressure Sensors

**DOI:** 10.3390/mi13050738

**Published:** 2022-05-06

**Authors:** Yuanjie Wan, Zhiwei Li, Zile Huang, Baofa Hu, Wenlong Lv, Chunquan Zhang, Haisheng San, Shaoda Zhang

**Affiliations:** 1 School of Electronic Science and Engineering, Xiamen University, Xiamen 361005, China; wanyuanjie@stu.xmu.edu.cn (Y.W.); lizhiwei2021@stu.xmu.edu.cn (Z.L.); 2 Pen-Tung Sah Institute of Micro-Nano Science and Technology, Xiamen University, Xiamen 361005, China; huangzile@stu.xmu.edu.cn (Z.H.); hubaofa@stu.xmu.edu.cn (B.H.); lwl1980@xmu.edu.cn (W.L.); cqzhang@xmu.edu.cn (C.Z.); 3 Shenzhen Research Institute of Xiamen University, Shenzhen 518000, China; 4 Cofoe Medical Technology Company Limited, Shenzhen 518100, China

**Keywords:** capacitive pressure sensors, MEMS, self-packaging, Si–glass bonding

## Abstract

This paper reports a MEMS capacitive pressure sensor (CPS) based on the operating principle of touch mode. The CPS was designed and fabricated using wafer-level self-packaged MEMS processes. The variable capacitance sensing structure was vacuum-sealed in a cavity using the Si–glass anodic bonding technique, and the embedded Al feedthrough lines at the Si–glass interface were used to realize the electrical connections between the parallel plate electrodes and the electrode pads through Al vias. The optimal design of the CPS structure was performed to trade-off the performance and reliability using finite element simulation. The CPS based on a circular-shaped diaphragm with a radius of 2000 µm and a thickness of 40 µm exhibits good comprehensive performance with a sensitivity of 52.3 pF/MPa and a nonlinearity of 2.7%FS in the pressure range of 100–500 kPa when the ambient temperature is less than 50 °C.

## 1. Introduction

Pressure sensors, as popular sensors in modern industrial society, are widely applied in industrial automation processes, aerospace and astronautical engineering, the petrochemical industry, deep-sea exploration, etc. [1,2,3,4,5]. Pressure measurement systems use different sensing principles, for example, piezoresistive, piezoelectric, and capacitive principles. Piezoresistive pressure sensors (PPSs) have the advantages of good linearity, high sensitivity, and a large pressure range; however, they are affected by temperature variations. Piezoelectric pressure sensors have a wide bandwidth, but no response at constant pressure. Capacitive pressure sensors (CPSs) are considered to have highly sensitive and robust structures. However, its output is nonlinear with respect to external pressure variation, which limits its application [6].

In the past decade, with the appearance and development of tire pressure monitoring systems (TPMSs), CPSs have been given more attention for application in TPMSs owing to the increasing need for devices with low and even no power consumption. Although battery-powered TPMSs based on PPSs are still irreplaceable in the current market, the power consumption of the system is unsatisfactory for a required lifetime of more than 10 years. CPSs have been verified experimentally to realize the pressure measurement and readout in the passive wireless monitoring of automobile tires [7,8,9,10,11]. This means that CPSs have a chance to become good substitutes for the well-known PPSs in TPMS.

A touch-mode CPS has been developed to reduce the nonlinear characteristics of CPSs [6,12,13,14,15]. The sensing diaphragm was designed to operate in a region where it touched the substrate mechanically. As a result, the output capacitance is nearly linear with the variation in external pressure. The robust structure enables it to withstand large overload pressures in harsh industrial environments [6]. To meet the application requirements of TPMSs, the sensors should support cars and light trucks with pressure ranges of 100–500 kPa and 100–900 kPa. It is difficult to achieve a CPS capable of meeting the requirements of multiple parameters in a TPMS, such as a high-pressure range, high sensitivity, good linearity, and high reliability.

In this work, a touch-mode CPS was designed and fabricated using a wafer-level self-packaging fabrication technique to improve the linearity and sensitivity of CPSs in a high-pressure range. A Si–glass bonding technique rather than the conventional Si–Si bonding technique was used to fabricate the pressure diaphragm, which can increase the yield of devices as well as long-term hermeticity [3]. The design, simulation, fabrication, and device characterization are presented and analyzed.

## 2. Design, Modeling and Simulations

**Figure 1:**
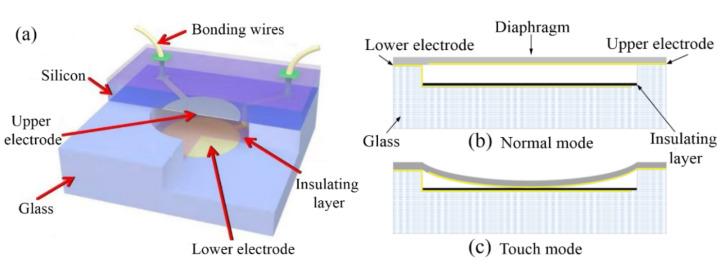
Schematic 3D structure of a self-packaged CPS (**a**) and its working principle in (**b**) Normal mode and (**c**) touch mode.

Figure 1a shows a schematic 3D structure diagram of a self-packaged CPS. A glass cavity was covered with a Si diaphragm to form a vacuum-sealed chamber. The upper electrode of the capacitor was located on the backside of the Si diaphragm, and the lower electrode of the capacitor was located at the bottom of the glass cavity and covered by a SiO_2_ insulator layer. Both the planar electrodes were vacuum sealed in a chamber. When a differential pressure is applied to the Si diaphragm, the diaphragm deforms in the normal mode when a small deflection occurs (Figure 1b). If the deflection of the diaphragm is large enough to contact the insulator layer, it results in touch-mode operation, as shown in Figure 1c.

**Figure 2:**
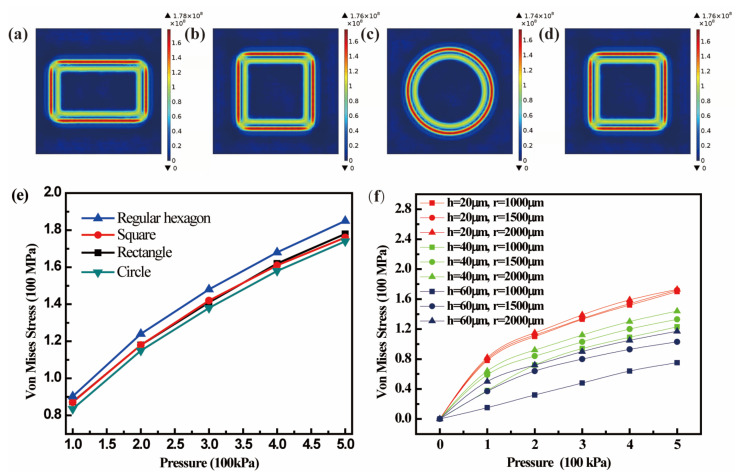
Simulated stress distributions on (**a**) rectangle, (**b**) square, (**c**) circular, and (**d**) regular hexagon diaphragms; (**e**) Simulated S-P curves based on different shapes of Si diaphragms with same area; (**f**) Simulated S-P curves based on circular Si diaphragm with same area and different thickness and radius.

To design the CPS, the reliability of the diaphragm was first considered. Using the same diaphragm area, the shape of the diaphragm had a significant influence on the stress distribution and maximum stress. Four different shapes of Si diaphragm (rectangular, square, regular hexagonal, and circular) with an area of ~12.56 mm^2^ and a thickness of 20 µm were modeled and simulated using the finite element method (FEM) in COMSOL Multiphysics^®^ software. Figure 2a–d show the simulated stress distribution on four different shapes of Si diaphragms under a pressure of 500 kPa. The Von Mises stress distribution was simulated to determine the location at which the maximum stress was generated. Figure 2e exhibits the dependence of the maximum stress on pressure (S-P) when using different shapes of Si diaphragm with the same area. It can be observed that the maximum stress is generally located at the clamped edge of the diaphragm, and the circular Si diaphragm has a minimum stress value with a continuous and consistent stress distribution at the clamped edge when compared with the other shapes of diaphragms, as shown in Figure 2c,e. This means that the circular diaphragm has higher reliability under the same work pressure and can withstand higher pressures than other diaphragms.

To optimize the design size of a CPS with a circular diaphragm, it is necessary to balance the trade-off between sensitivity and reliability. A large radius or small thickness can result in a higher sensitivity but lower reliability. Figure 2f shows the simulated S-P curves based on a circular Si diaphragm with the same area and different thickness and radius. It can be seen that the maximum Von Mises stress increased with a reduction in thickness and an increase in radius. Meanwhile, it was found that with an increase in the thickness, the effect of the radius on the stress is reduced. Indeed, the stress is insensitive to the radius when the thickness reaches 20 µm. Considering that the maximum stress in a 20 µm thick diaphragm is far less than the allowable stress of Si (500 MPa), 20 µm appears to be the optimum radius. However, a linear capacitance-pressure (C-P) output in the required work range is more important for practical sensing applications and should be further simulated to determine the optimum thickness and radius of the diaphragm. The effects of the radius and thickness of the diaphragm on the CPS performance can be obtained by calculating the capacitance with respect to the pressure-dependent deflection of the diaphragm.

For a touch-mode CPS with a circular diaphragm, the capacitance varies with the change in distance between the top and bottom electrodes. The capacitance of the CPS can be given by [15]:

(1) $$C=\int_{0}^{r}\frac{2\pi \varepsilon_{0}\varepsilon_{i}rdr}{t+\varepsilon_{i}\left(g-w\left(r\right)\right)}$$

where *ε_0_*, *ε_i_* are the vacuum and relative dielectric constants of SiO_2_, respectively, *t* is the thickness of the SiO_2_ insulation layer, and *g* is the cavity depth on the glass substrate. *w(r)* is the deflection of the Si diaphragm with respect to its touch radius *r* of the Si diaphragm. This can be expressed as [15]

(2) $$w\left(r\right)=\frac{12Pr_{0}{}^{4}\left(1-v^{2}\right)}{64Eh^{3}}*{\left[1-{\left(\frac{r}{r_{0}}\right)}^{2}\right]}^{2}$$

where *P* is the pressure on the diaphragm, and *ν* and *E* are Poisson’s ratio and Young’s modulus, respectively. *r_0_* and *h* are the radius and thickness of the Si diaphragm, respectively.

**Figure 3:**
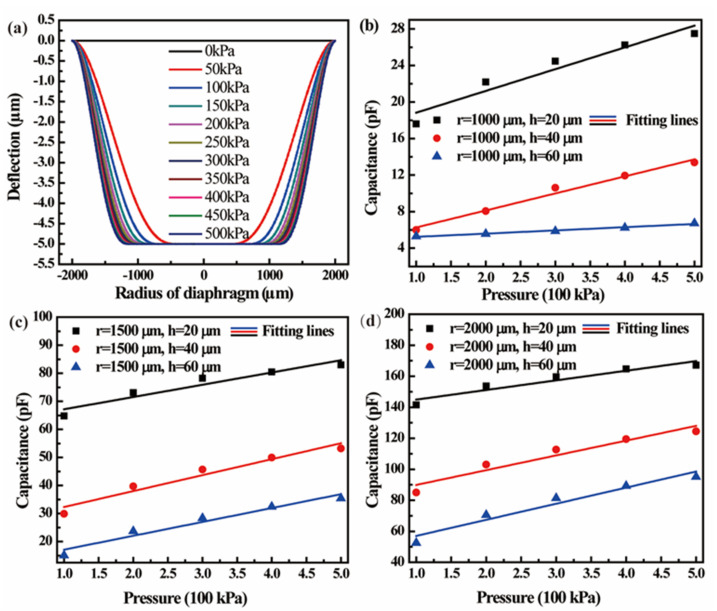
(**a**) Effect of pressure on deflection of diaphragm with radius of 2000 µm and thickness of 40 µm; C-P characteristics of CPSs with diaphragm radius of (**b**) 1000, (**c**) 1500, and (**d**) 2000 µm in different diaphragm thicknesses.

Figure 3a shows the simulated pressure-deflection (P-D) curves of a circular diaphragm with a radius of 2000 µm, a thickness of 40 µm, a cavity depth of 5 µm and a 1.8 µm thick insulating layer. It can be observed that the CPS is operated in the touch mode when the pressure is greater than 50 kPa. By Equations (1) and (2), the capacitance of the CPS can be calculated. Figure 3b–d exhibit the C-P characteristics of CPSs with diaphragm radii of 1000, 1500, and 2000 µm when the diaphragm thicknesses are 20, 40, and 60 µm, respectively. Table 1 presents the performance parameters of CPSs with diaphragms of different sizes. It is found that the sensitivity increases with an increase in radius, while the nonlinearity decreases with an increase in thickness. The 2000 µm@60 µm group exhibited the best performance parameters. Considering the requirement for a capacitance value that is as high as possible, the 2000 µm@40 µm group is suggested to be the optimum size.

## 3. Fabrication and Measurement of Devices

**Figure 4:**
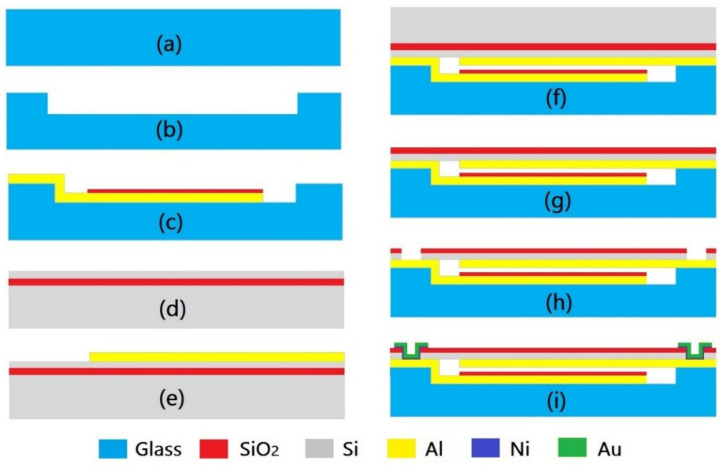
Self-packaged fabrication process flow of MEMS-based CPSs. (**a**) Glass wafer; (**b**) Etching cavity; (**c**) Depositing Al and SiO_2_; (**d**) SOI wafer; (**e**) Depositing Al; (**f**) Two wafer bonding; (**g**) Removing Si substrate; (**h**) Opening electrode windows; (**i**) Depositing Au electrode.

The devices were fabricated using microelectromechanical system (MEMS) technology. Figure 4 shows the MEMS process flow of CPSs based on the self-packaged fabrication technique [16]. The main fabrication processes are as follows:

(1) Fabrication of the cavity and lower electrode in a Borofloat 33 (BF33) wafer: An acid solution (HF:NH_4_F:H_2_O = 3: 6: 10) was used to etch the 7-µm-deep circular cavities in the BF33 glass wafer with Cr (10 nm)/Au (100 nm) as a mask (Figure 4b). To achieve an in-plane embedded electrode at the bonding interface, 200-nm-deep trenches were etched onto the surface of the glass wafer. For each device, two trenches were designed to accommodate the Al feedthrough lines connecting the upper and lower electrodes of the variable capacitor. Next, a 200-nm-thick Al film was deposited on the bottom of the cavity and filled into the trenches of the lower electrode through a sputtering and lift-off process, after which a 1.8-µm-thick SiO_2_ insulating layer was grown and covered the Al electrode through the plasma-enhanced chemical vapor deposition (PECVD) technique. Following the above step, the SiO_2_ insulating layer was patterned using an inductively coupled plasma (ICP) etching process (Figure 4c).

(2) Fabrication of the pressure diaphragm and the upper electrode: An n-type (100) silicon-on-insulator (SOI) wafer with a 40-µm-thick device layer and a 0.5-µm-thick buried SiO_2_ layer was used to fabricate the pressure diaphragm. To fabricate the upper electrode and feedthrough line, a 200-nm-thick Al film was deposited on the SOI wafer through a sputtering and lift-off process (Figure 4e). Next, the SOI wafer and BF33 wafer were anodic-bonded together (Figure 4f). The bonding temperature and voltage were set to 360 °C and 800 V for 30 min, respectively [17,18]. Meanwhile, an extra 300-N pressure force was applied to the bonding wafer for the Al feedthrough line embedding in the bonding interface. After anodic bonding, the Si substrate layer of the SOI was removed by wet etching in 35% KOH at 80 °C to form a Si diaphragm (Figure 4g). In order to maintain the vacuum, a getter material will be packaged in the pressure chamber in practical engineering applications.

(3) Fabrication of the electrode vias and Pads: ICP etching was used to fabricate the electrode vias in the Si diaphragm, with the embedded Al layer as the etching stop layer (Figure 4h). Next, a Ni/Au (50/500 nm) layer was deposited in the vias to form an Al/Ni/Au electrode pad through a sputtering and lift-off process (Figure 4i).

**Figure 5:**
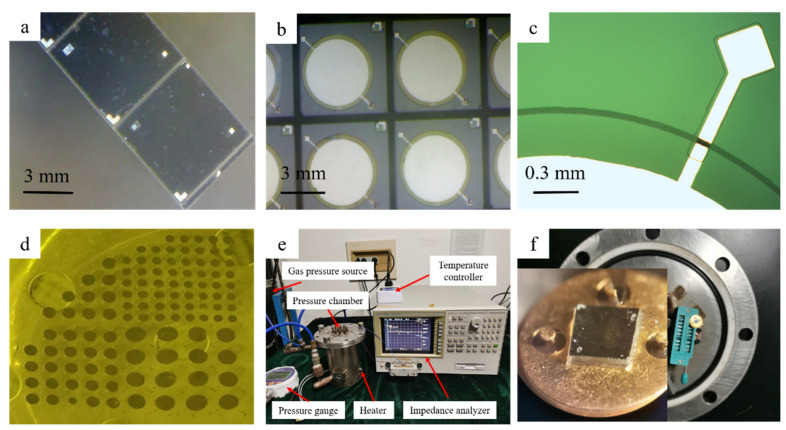
Photos of (**a**) the fabricated devices and the partial structures of (**b**) lower electrode, (**c**) feedthrough line and (**d**) insulating layer in the glass cavities; Photos of (**e**) the measurement system and (**f**) the mounting location of sensor and the device packaged in TO39 socket.

Figure 5a shows the photo of fabricated devices. Figure 5b exhibits the photo of the fabricated electrode structure on a glass wafer, and Figure 5c displays the enlarged photo of the Al feedthrough line connected to the lower electrode on the glass cavity. Figure 5d shows the PECVD SiO_2_ insulating structures deposited on the glass cavities with different sizes.

The performance of the sensors was measured using a C-P test system (Figure 5e). In this system, the sensors were placed in the pressure chamber and fixed at the back of the cap of the pressure chamber, which was mounted in the TO39 socket, as shown in Figure 5f. A 20 MPa high-pressure nitrogen gas cylinder was used as the pressure source, and a pressure-reducing valve was used to control the pressure in the chamber. A high-precision pressure gauge was used to determine real-time pressure values. A heater connected to a temperature controller is used to change the ambient temperature in the chamber. The capacitance of the sensors was measured using an impedance analyzer.

## 4. Results and Discussion

**Figure 6:**
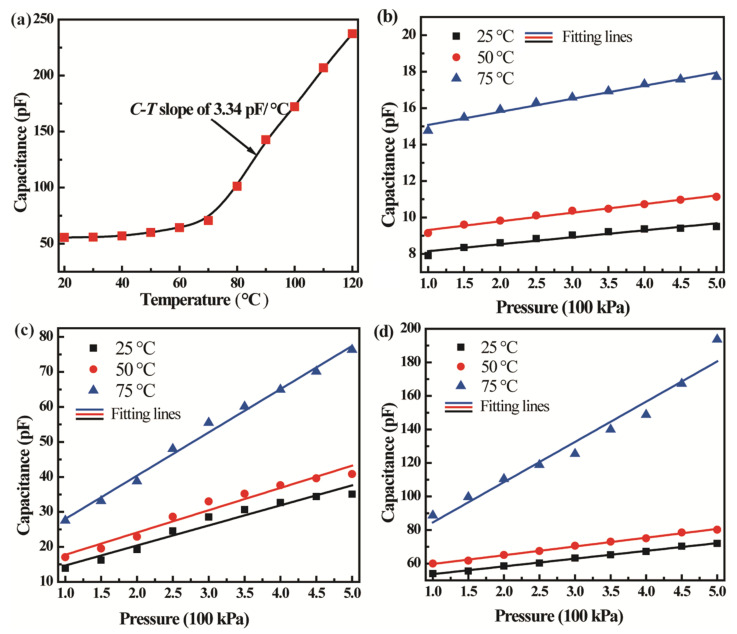
(**a**) C-T characteristic of CPS with diaphragm radius in 2000 µm; C-P characteristics of CPSs with diaphragm radius of (**b**) 1000, (**c**) 1500, and (**d**) 2000 µm at the ambient temperature of 25, 50, and 75 °C, respectively.

The C-T characteristics of the CPSs were first investigated to determine their working temperature range. Figure 6a shows the C-T characteristics of the CPS with a diaphragm radius of 2000 µm in a standard atmosphere. It can be seen that the temperature change has little influence on the capacitance variation of the device when the working temperature is less than 50 °C. With an increase in the working temperature from 50 °C to 70 °C, the capacitance increases slowly. When the temperature is greater than 70 °C, the capacitance rapidly and linearly increases with a C-T slope of 3.34 pF/°C. It is considered that the elastic modulus of the Si diaphragm is reduced with an increase in temperature, thereby resulting in an increase in capacitance.

Figure 6b–d show the C-P characteristics of CPSs with diaphragm radius in 1000, 1500, and 2000 µm at temperatures of 25, 50, and 75 °C, respectively. All CPSs display pressure-dependent capacitance characteristics, proving the availability and reliability of CPSs fabricated using self-packaging MEMS processes. It can be seen from C-P characteristics that the temperature has an influence only on the intercept of C–P curves when the temperature is less than 50 °C. As the temperature is larger than 50 °C, both the intercept and slope will be affected. Although the temperature influence on CPS@1000 µm is minimum, the low sensitivity limits its application. Table 2 exhibits the parameters of CPSs with different radii of diaphragms. In comparison with the CPS@1500 µm, the CPS@2000 µm has better performance with a sensitivity of 52.3 pF/MPa and a non-linearity of 2.7%FS when the ambient temperature is less than 50 °C. In comparison with simulated results, the measured results are better in non-linearity with relatively low sensitivity. It is suggested to increase the work temperature range by adding a stress compensation layer on the diaphragm.

**Figure 7:**
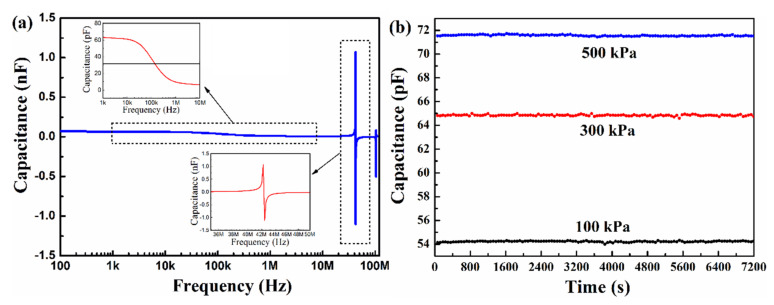
(**a**) C-F and (**b**) C-T characteristics of CPSs with diaphragm radius in 2000 µm at the ambient temperature of 25 °C.

Figure 7a shows a capacitance-frequency characteristic of CPSs with a diaphragm radius of 2000 µm. It is observed that the resonance and anti-resonance peaks are at ~42.2 MHz, as shown in the inset of Figure 7a, and the CPSs demonstrate a very flat C-F response characteristic in the low-frequency region (≤20 KHz). In the high-frequency region, the low capacitance value will lead to a low capacitance-change-rate (CCR) and thus a low sensitivity of devices. Figure 7b shows a long-term capacitance-time (C-T) characteristic of CPSs with a diaphragm radius of 2000 µm at the ambient temperature of 25 °C and under different pressures. It can be seen that the capacitance values of CPSs under pressures of 100, 300, and 500 kPa remain steady (CCR ≤ 0.6%) in a long-term measurement of 7200 s, respectively, implying good vacuum-sealed reliability of devices.

## 5. Conclusions

In this work, a MEMS CPS based on the operating principle of the touch mode was presented. CPSs with diaphragms of different shapes and sizes were modeled and simulated using FEM in the COMSOL platform. It is suggested that the circular diaphragm has a better balance between performance and reliability. Considering the requirement for a capacitance value that is as high as possible, a radius of 2000 µm and thickness of 40 µm was determined to be the optimum size. CPSs were fabricated using wafer-level self-packaged MEMS processes. The variable capacitance sensing structure was vacuum-sealed in a chamber using the Si–glass anodic bonding technique, and the embedded Al feedthrough lines at the Si–glass interface were used to realize the electrical connections between the parallel plate electrodes and the electrode pads through Al vias.

In summary, the measured results show that the CPSs with optimum size have good comprehensive performance with a non-linearity of 2.7%FS and a sensitivity of 52.3 pF/MPa in the pressure range of 100–500 kPa when the ambient temperature is less than 50 °C. The CPSs exhibit a good low-frequency characteristic with a bandwidth of 20 kHz, and a long-term C-T measurement also verifies good vacuum-sealed reliability of devices. It is suggested that the working temperature range can be increased by adding a stress compensation layer on the diaphragm.

## Figures and Tables

**Table 1:** Performance of CPSs with different sizes of diaphragms.

Parameters	Values								
Radius (μm)	1000			1500			2000		
Thickness (μm)	20	40	60	20	40	60	20	40	60
Sensitivity (pF/MPa)	36.8	18.7	3.5	43.7	56.9	49.5	62.1	95.3	103.9
Non-linearity (%FS)	12.9	8.2	5.8	13.6	10.5	10.3	13.6	12.7	10.7

**Table 2:** Parameters of CPSs with different radii of diaphragms.

Parameters	Value								
Radius (μm)	1000			1500			2000		
Temperature (°C)	25	50	75	25	50	75	25	50	75
Sensitivity (pF/MPa)	3.8	4.7	7.2	57.3	63.7	124.1	46.2	52.3	240.1
Non-linearity (%FS)	15.9	9.0	10.8	11.1	9.8	5.6	2.4	2.7	13.6
